# Owners’ Knowledge and Approaches to Colic in Working Equids in Honduras

**DOI:** 10.3390/ani11072087

**Published:** 2021-07-13

**Authors:** Isabella Wild, Sarah Freeman, Daniela Robles, Dennis Matamoros, Maverick Ortiz, Jonathan Rodriguez, John Burford

**Affiliations:** 1 World Horse Welfare, Anne Colvin House, Snetterton, Norwich NR16 2LR, UK; 2 School of Veterinary Medicine and Surgery, Sutton Bonington Campus, University of Nottingham, Sutton Bonington, Loughborough LE12 5RD, UK; sarah.freeman@nottingham.ac.uk (S.F.); john.burford@nottingham.ac.uk (J.B.); 3 Equinos de Honduras, Barrio Tamarindo, 500 Metros al Oeste de Antiguo Local del Colegio Adventista, Choluteca 51101, Honduras; drobles@equhs.org (D.R.); dnnisbnavidez@gmail.com (D.M.); maverick_ortiz8a@hotmail.com (M.O.); jrodriguez@equhs.org (J.R.)

**Keywords:** working equids, equine nutrition, equine welfare, equine colic, One Welfare, veterinary education, horse husbandry

## Abstract

**Simple Summary:**

There are approximately 275,000 equids in Honduras, with many providing tractional assistance to human lives and livelihoods. The aim of this study was to define the role and improve the welfare of working equids through obtaining a baseline understanding of equine colic or ‘abdominal pain’ in the Choluteca region of Honduras. The objectives were: to explore owners’ current understanding of colic in Honduras through questionnaires and to identify knowledge gaps where educational materials could benefit owners and working equid welfare. Ninety-three verbal questionnaires were conducted with owners from eleven different communities in the Choluteca region on their use of working equids and knowledge and treatment of colic. Additional context was gained through observations and verbal questionnaires with three veterinary practitioners and eight agricultural pharmacy (agrovet) store owners. Equids played a role in firewood collection, the transportation of people, and carrying crops. Most owners with previous experience of colic had treated it themselves, typically using their own remedies. Owners with prior knowledge or experience of colic expressed concern about the condition. Across all stakeholders, knowledge and understanding of colic varied, and access to evidence-based treatments for owners was very limited. Areas for improving education on equine colic were highlighted in this study.

**Abstract:**

In Honduras, many families are reliant on working equids in their daily life. The aim of this study was to evaluate knowledge about, and approaches to colic used by owners of working equids in Choluteca, Honduras using a phenomenological approach. Semi-structured, verbal questionnaires were conducted with ninety-three owners from eleven different communities in the Choluteca region on equid horse owners’ knowledge of colic and treatments. Additional context was gained through observations and verbal questionnaires with three veterinary practitioners and eight agricultural pharmacy (agrovet) store owners. Working equids were commonly used for firewood collection 31% (40/126), transportation 24% (30/126), and carrying crops 13% (17/126). Thirty-eight percent of owners (35/92) said they did not know what colic was, 27% (24/89) could not name any clinical signs, and 46% (42/92) could not name any causes. Most owners with previous experience of colic had treated it themselves 79% (53/67), typically using herbal remedies. Colic was a major concern for owners of working equids who had prior experience or knowledge of the condition. Knowledge and understanding of colic varied, and access to evidence-based treatments was very limited. The findings will be used to inform the development of educational resources on colic in working equids.

## 1. Introduction

The population of working equids in low- and middle-income countries (LMICs) is estimated at approximately 100–112 million, a figure which is likely underestimated [1,2]. ‘Working equids’ are horses, donkeys, and mules that work alongside humans, contributing to income generation (direct and indirect) and the improvement of daily lives and livelihoods [3]. Working equids are estimated to benefit 600 million people worldwide, through the daily tasks they contribute to [4,5]. Colic (abdominal pain) is a major welfare concern in the general equid and working equine population [6,7]. The underlying diseases causing colic (and its identification, treatment, and prevention) are influenced by a range of different factors. There are few published studies on colic in working equids, and these are limited to studies from Africa and India [6,7,8,9,10].

Working equids are relied upon by often impoverished owners [11] and are employed in a number of physically demanding jobs in tough environmental conditions [4,12,13,14]. Communities which rely on working equids are often remote, with a lack of access to facilities, education, veterinary services, and financial resources, which can have a knock-on effect on equid welfare [15,16]. Poor welfare not only affects the animal but also has an impact on human dependents, with days lost in work if the animal is unable to function to its full potential. The role of working equids makes a significant contribution to many of the United Nation’s Sustainable Development Goals (SDGs) [4], in particular: strengthening livelihoods, improving access to water, building resilience, productive farming, empowering women, and enabling education. Results from one recent study showed that improving animal welfare and improving human welfare through achieving the SDGs go hand-in-hand [17], and this is considered a One Welfare approach [18,19].

Honduras is a Spanish-speaking country in Central America, where 48.3% of the population lives in poverty [20]. World Horse Welfare, a United Kingdom based Non-Governmental Organization (NGO), partners with a charity called Equinos de Honduras (EQUHS) based in Choluteca, Honduras [21]. Firewood collection is one of the most common uses for working equids to generate income, and equids work on average five hours a day, for six days per week, travelling up to fifteen kilometers daily [22]. Income for working equid owners is also considered to be low in this area [22]. There are approximately 275,000 equids in Honduras [1] (the population for the Choluteca region is unknown), with approximately 3750 equids and 2600 owners registered to the welfare project with EQUHS in Choluteca. There is minimal published research on disease in equids in Honduras [23,24] and no published research on equine colic.

Colic, or ‘abdominal pain’, is regarded as the most common and important emergency condition in the horse [25,26]. Colic has a relatively high incidence, and coupled with its significant mortality this condition poses a substantial welfare concern in equids. In the United Kingdom (UK), a campaign centered on the recognition of colic in equids has been developed at the University of Nottingham. This involved the development of evidence-based educational resources for both owners and veterinary practitioners in partnership with the British Horse Society and World Horse Welfare [27,28]. Whilst these resources were aimed at the pleasure horse population (e.g., horses ridden as a hobby, or kept for companionship), with data mainly collected from the UK, there is an opportunity to broaden this campaign, learn from other countries worldwide, and address the welfare issues faced with equine colic within a wider population.

The aim of this project was to define the role and improve the welfare of working equids through obtaining a baseline understanding of equine colic in the Choluteca region of Honduras.

The objective of this project was to first explore owners’ current understanding and experiences of colic in Honduras through verbal questionnaires and field notes. The second objective was to identify knowledge gaps where educational materials could benefit owners and working equid welfare.

## 2. Materials and Methods

The study was reviewed and approved by the School of Veterinary Medicine and Science (SVMS), University of Nottingham, Ethics Committee (2198 180131). The study explored the role and work of the equids and their owners’ knowledge, understanding, and approaches to colic. The study was based on an interpretative paradigm using a phenomenological approach. In-depth interviews were not possible due to the local environment and language barrier. The researcher spent three weeks visiting working equid communities in Honduras, working alongside the local equine charity, conducting verbal questionnaires with local horse owners through a translator, and observing and interviewing staff and volunteers involved in the local charity. The researcher was a qualified veterinary surgeon, with experience of owning and handling horses in the UK, and previous research experience of qualitative and quantitative methodologies and analyses from an MRes on disseminating evidence to veterinary practitioners in the UK.

### 2.1. Questionnaires

The questionnaires were developed in the UK based on previous colic questionnaires in the UK, Morocco [6,29], and background reading in this area [22,30,31]. The format used semi-structured, open-ended questions, with pre-defined prompts and further follow-up queries. For four questions, multiple answers were encouraged, these are denoted with an asterisk in the Supplementary Items, File S1. The proposed questionnaire was reviewed by two academic researchers at SVMS, and piloted with the EQUHS team; undergoing adaptation following feedback. The owner questionnaire is available in Supplementary Items, File S1. All questions were optional; however, answers to each question were encouraged, unless it was impossible for the owner to comment.

Convenience sampling of participants was used, recruiting from attendees of the EQUHS community visits for three weeks during June/July 2018 in the region of Choluteca, Honduras. The communities were a mixture or rural and urban (Supplementary Items, Table S1) and had all previously been involved in EQUHS community visits. The sole eligibility criterion for participation was that the respondent was an owner/keeper of a working equid(s). Informed consent was obtained from all participants, following verbal delivery of the participant information and consent document. The consent of the participant was recorded by signature or in some cases verbal consent was given if the participant was unable to write. Questionnaires were also undertaken with a small number of other stakeholders, including veterinary practitioners (a vet from EQUHS, a newly qualified veterinary practitioner, and a university veterinary lecturer) and ‘agrovets’ using a separate verbal questionnaire. ‘Agrovet’ refers to a farming supply store, commonly found in Central America, which sells animal feed, seed, fertilizer, farming equipment, and veterinary products over the counter. The questionnaires were undertaken verbally using a bilingual Spanish translator, who was a newly qualified veterinary practitioner.

### 2.2. Field Notes and Observations

These were undertaken on a daily basis at the EQUHS office and from reports on observations from visiting the different paraprofessionals and communities and from discussions with staff at the headquarters.

### 2.3. Data Analysis

Verbal questionnaires were recorded, transcribed, and entered into Microsoft Excel. All data were anonymized, grouped via community, and stored in password protected files. Descriptive statistics were calculated for all data (frequency and percentage). The semi-structured verbal questionnaires produced short answers (mostly 1–3 words) which were categorized and grouped into themes as appropriate. Where applicable, categorical data were derived from grouping numerical data, e.g., hours per day and days per week worked if the format of the response varied. Colic clinical sign categories were consolidated according to the British Horse Society REACT acronym (Supplementary Items, Figure S1) [27], and colic treatment categories were also grouped for further analysis. The REACT acronym was developed as part of a colic educational campaign in the UK, with the acronym spelling out different signs of colic: Restless or agitated, eating less or droppings reduced, abdominal pain, clinical changes, tired or lethargic.

## 3. Results

### 3.1. Equid Owners

A total of 93 verbal questionnaires were carried out in 11 different communities (Table 1). These lasted three to twelve minutes. Some questions on the original questionnaire were found to be redundant and were discontinued after the piloting and initial verbal questionnaires. There was some encryption on all four of the questionnaires from Santa Maria, which meant that not all results from all of the questions could be analyzed.

### 3.2. Demographics

Ninety-three percent of owners interviewed were male (87/93) and 71% owned 1–2 equids (65/92), 22% owned 3–4 equids (20/92), 5% owned 5–6 equids (5/92), and 2% owned >7 equids (2/92). The majority owned horses 86% (80/93), with a few owners also owning donkeys and mules. The most common types of work were collecting firewood 31% (40/126), people transportation 24% (30/126), and carrying crops 13% (17/126). Most commonly, 59% (54/92) of equids were involved in one type of work, with 35% (32/92) undertaking more than one type of work, and 7% (6/92) not working. Most commonly, equids worked 3–5 h a day (40%) (36/90), followed by 31% working 6–9 h a day (28/90), and 26% working 0–2 h a day (23/90). Thirty-three percent (30/90) of owners reported that equids worked for 6–7 days a week, followed by 0–1 days and 2–3 days a week both at 23% (21/90), and 16% working 4–5 days a week (14/90).

### 3.3. Community Profiles

Field notes taken from the communities visited demonstrated differences including proximity to larger towns, climate, infrastructure, types and uses of equids, and responses given in the questionnaire. Summary data is presented in Supplementary Items, Table S1 and a brief description is displayed in Table 1.

### 3.4. Recognition and Understanding of Colic

When asked about their understanding of colic, 62% of owners knew what colic was (57/92) and 73% (65/89) could name one or more clinical sign, with the most common being: ‘rolling’ 32% (58/181), ‘could not stand’ 10% (19/181), ‘lying down and getting up’ 9% (17/181), and ‘running’ 9% (16/181). Clinical signs recognized by equid owners are documented in Table 2. Most commonly, owners could not name any cause (46%) (42/92), 40% could name more than one cause (37/92), and 14% could name one cause (13/92). The most common causes mentioned were parasites 27% (25/91), nutritional/dietary 22% (20/91) (type of food *n* = 6, bad/contaminated food *n* = 5, too much carbohydrate *n* = 3, eating cabbage *n* = 2, change of diet *n* = 1, new grass *n* = 1, too much food *n* = 1, no food *n* = 1), eating rubbish/plastic bags 16% (15/91), too much work 7% (6/91), and plant toxin/poison 7% (6/91).

Seventy-one percent of owners had previous experience of colic (65/92), with 79% treating with their own remedies (53/67). Treatments recorded are tabulated in Table 3. The most commonly used treatments were: guasimo 21% (34/162), garlic 10% (17/162), curarina 6% (10/162), vinegar 6% (10/162), and unknown injection/pill 6% (9/162). Owners reported that most working equids had recovered from colic 80%(52/65), 17% (11/65) did not recover, and 3% (2/65) that some recovered and some did not. What happened to the equids that did not recover was unclear, but from the understanding of the local veterinary practitioner and field observations, it was likely that the equids just died. When asked ‘When would you use the vet?’ 25% (23/93) said they would use the ‘agrovet’, 22% (20/93) would treat first and use the veterinary practitioner if their own treatments did not work, 17% (16/93) would use the veterinary practitioner when the equid was unwell, 13% (12/93) only when the veterinary practitioner/‘brigades’ visit the community, and 11% (10/93) would not use a veterinary practitioner. Brigades refer to the ‘Equitarian’ projects from the United States, which provide training and veterinary work to working equid communities on an annual basis [32].

### 3.5. Husbandry

Forty-five percent (41/91) of equids were reported to have access to water all day, with 32% (29/91) twice a day. The water source was from the river for 51% (46/91) of equids and a well for 42% (38/91). Sixty-two percent were fed only grass (57/92) and 73% had changes in feed in the summer and/or with a change in workload (67/92). Most equids were not constrained when not working 75% (69/92) but 96% of owners said their equids did not have access to rubbish when unattended (88/92). Seventy-one percent owners reported their equid had been treated previously with anthelmintic (65/92) and 81% (74/91) reported their equid had not received any dental care.

### 3.6. Observations and Questionnaires with Veterinary Practitioners and Agrovets

There are some key differences between the UK and Honduras with regards to veterinary practice. The UK is used as a comparator since this is where the original educational resources were developed. In Honduras, ‘Agrovets’ sell veterinary pharmaceuticals alongside various other farming tools and equipment. Many prescription-only drugs in the UK are easily accessible via the agrovet in Honduras. Some ‘controlled drugs’ in the UK (requiring strict storage, prescription, dispensing, and destruction (VMD)) for example ketamine, are available for anyone to buy. Antibiotics were also found to be freely available such as oxytetracyline and cephalexin, which raises concern regarding antimicrobial resistance if there is not control, education, or guidance with these drugs. Conversely, some drugs commonly used in the UK are banned or unavailable, e.g., butorphanol, an opioid for sedation analgesia is banned and detomidine an alpha-2 agonist, used often alone or in combination with butorphanol for sedation and analgesia is unavailable, but an overseas veterinary group supply EQUHS when they visit.

A small number of verbal questionnaires were carried out with qualified veterinary practitioners (3) and agrovets (8) and were analyzed using thematic analysis with an inductive approach. Themes identified included: both groups recognizing colic as a welfare concern, poor training in equine veterinary studies for veterinary practitioners, equid owners visiting the agrovet before the veterinary practitioner, differences in drug choices between veterinary practitioners and agrovets and fair/varying knowledge on signs/causes of colic. Individual veterinary practitioners spoke about different causes of colic. One veterinary practitioner at EQHUS reported seasonal changes in colic incidence, with an increased incidence of colic in the summer months, due to dehydration, increased feeding of concentrates, and equids scavenging for food and ingesting plastic bags. Both veterinary practitioners and agrovets mentioned contaminated food or fungal toxins as possible causes of colic. The veterinary practitioners reported the availability of training of equine veterinary practitioners to be poor, with more focus on other areas at university and no further training. The veterinary practitioners interviewed showed varying, but mostly good, knowledge/experience of colic, and a larger survey would be useful to understand their levels of knowledge and experience. It was found that owners would only bring the equids to the agrovet or veterinary practitioners at the very last stages and that euthanasia was infrequent. The agrovet would usually be the first point of call.

There were differences in the treatments typically administered by the two groups. The treatments used by veterinary practitioners were: analgesia/flunixin (3), fluids (3), spasmolytic (2), oil (2), opioid (2), alpha-2 agonist (1), lidocaine infusion (1), electrolytes (1), and enema (1). The treatments given by agrovets were ranked spasmolytic (6), diuretic/furosemide (4), fluids (4), analgesia/NSAID/flunixin (3), beer (1), dextrose (1), and vitamins (1). The veterinary practitioners and agrovets involved had a fair knowledge on the presentation of colic, most frequently listing obvious signs such as rolling; with only one agrovet unable to list any signs of colic. Most veterinary practitioners and agrovets reported that donkeys and mules show the same clinical signs as horses, although some reported that these species do not suffer from colic.

## 4. Discussion

Working equids are essential for everyday life for many in Honduras. In this study, the most common tasks undertaken by equids (firewood collection, transportation, and carrying crops) highlighted their vital importance to local communities and the economy. There was poor owner knowledge around colic signs and treatment. Most commonly, owners would use their own herbal treatments. There were variations in the equids’ access to water and roughage, with some having seasonal changes in feeding patterns. Following using their own treatments, the agrovet was often the next source of information and treatment for owners, and access to qualified veterinary practitioners was difficult. There was limited equine training/resources available for agrovets and veterinary practitioners in Honduras.

### 4.1. Demographics

The high proportion of male equid owners and agrovets reflects cultural roles in Honduran society, where the men do most of the work for income, including with animals. The few women owners who did participate were mainly not owners, but were looking after the animal for their spouse or male relative. This reflects the demographics of working equid ‘owners’ worldwide shown in many other working equid studies [6,15,33], although the ‘owner’ is a different role to ‘caregiver’ or ‘user’ [34,35].

Gender roles were not explored in detail in this study, but women are still likely to be involved in care and use of the equids. In another study in Central America [36], it was found that whilst women were not considered the ‘owner’, equids were often used by women to help with household chores, and over 90% of women in this study reported that they were responsible for primary care of the equid, including providing food and water provisions. These women also reported they did not attend training sessions arranged by World Horse Welfare since they were not the ‘owner’. In many cases they were unable to use the equids for transportation; however, over 50% were interested in learning more about diseases. This knowledge should be carefully considered when targeting educational resources on equine colic and accessing these primary caregivers, as when the animal is not working, it may be women who are responsible for animal husbandry. This partnership of women and working equids is reflected in studies across the world [35].

### 4.2. The Importance of Equids to Owners

As displayed in the community profiles, the versatility of equids means they are used for different tasks depending on their location, which is reflected in other studies [12,37]. The most common roles across all communities were firewood collection, transportation, and carrying crops; however, in the more remote areas, equids are essential for transportation due to the limited infrastructure and challenging terrain [12]; in the town for rubbish collection [38,39]; in the hottest areas for collecting and selling water [4]; and in specific dairy communities for carrying milk and cattle work [4,35]. There was variation in how frequently equids were worked; however, most commonly equids were used for significant periods of time (3–5 h/day), most days of the week. These working patterns are quite typical for working equids [33,37] and indicate their important role in their communities. Most commonly, owners relied on just one or two equids. The days lost in work due to illness, injury, or death, are likely to have a significant effect on the owner and their communities. This is a concern that has been voiced by equine owners and keepers in other studies [35,36].

### 4.3. Care and Husbandry of Working Equids, and Reducing the Risk of Colic

Management of equids is closely linked with some underlying causes of colic [40,41], so it was important to determine current husbandry practices. Causes and risk factors of colic were poorly understood by owners, with nearly half of owners unable to name a cause. In the literature and anecdotally, a wide range of causes are recognized in pleasure horses, including feeding and watering, parasite control, dentition, grass growth, stabling, exercise, natural predisposition, stress, windsucking/crib-biting, sedation, and general anesthesia [27,41]. The environment and management of working equids is very different. Some of the previously described factors are not applicable to the equids in this study, e.g., stabling and general anesthesia. Other factors play an important role in the development of colic in working equids, such as access to water and type of food [30].

In this study in Honduras, most horses accessed water from a river or well. Although the river water could be clean, in some areas there was contamination due to gold mining. Based on this, a key aspect of future education would be to advise owners to give clean, fresh, non-contaminated water. Just under half of equids were reported to have access to water all day, similar to another study in Chile [42]. Despite this, one veterinary practitioner reported that in the hotter months, equids often showed signs of dehydration, which has negative impacts on welfare [43]; may contribute to certain presentations of colic, such as impactions [27]; and in severe cases can lead to weakness, recumbency, and death [44]. Dehydration is a common finding in the working equid population [44,45], where equids are working hard in hot climatic conditions. In a colic study in Morocco, it was found that 14% of equids were offered water once a day and 2% every other day [6]. In a study in working equids in India, the most common type of colic diagnosis was impaction (65%), which the author considered likely due to dehydration and lack of low digestibility coarse feed [9]. Again, this highlights a key area for owner advice and welfare recommendations.

Approximately a quarter of participants reported diet and nutrition as contributing factors of colic; this was lower than expected, as diet is an important risk factor for equine colic. However, many owners fed only grass to their equids, which has greater digestibility and may be important for colic prevention [27,40,41]. Food that is contaminated or has fungal toxins was another colic cause given by some owners, veterinary practitioners, and agrovets, which has been documented in other studies [46,47]. Most owners changed their animal’s feed according to workload and forage availability, similar to findings from other working equid populations [42]. From an educational perspective, important aspects of diet management are that feed should be changed gradually and the animal should receive sufficient high quality roughage to prevent colic episodes [27,40,41,48]. Feeding increased whole-grain corn (typical in these equids’ diets) has been shown to increase colic incidence [49], and type of feed has been shown to increase the risk of colic in working equids in Egypt [8]. EQUHS reported they have recently started growing specific species of grass in fields, which is more resistant to arid conditions, which should help provide quality roughage to working equids in the hotter months, when they lose body condition.

Working equids can suffer from foreign body impactions from inorganic material, particularly when food is scarce [10,30]. In this study most owners said their equids did not have access to rubbish when unattended, but with 75% of owners reporting their equids to be free roaming, e.g., kept loose, without a fence or corral and allowed to roam the streets unsupervised, where plastic bags/rubbish are ubiquitous, this is still a risk. In a study in Morocco only 25% of equids were kept loose and able to feed unsupervised during the time spent the time resting [6]. With the differences in responses to these questions with owners reporting access to rubbish being low but a high percentage being free roaming, there was perhaps some misunderstanding with these questions, or a lack of relation from the owners between keeping an equid loose and having access to rubbish. However, looking at the data as a whole, colic caused by foreign body impaction is a risk in this area and again should be considered as a focus for future advice/education.

Opinions on routine preventative health care varied. The majority of owners used anthelmintics in their animals, and it was clear they were aware of parasites being a possible welfare problem. All of the owners were at the community visit, during which treatment for endoparasites was administered, so there will be selection bias. Only a minority had their equid’s teeth rasped, which had all been done in the communities near the clinic and by overseas veterinary practitioners. Poor teeth may lead to poor mastication and an increase in colic and impactions lower down in the gastro-intestinal tract [50,51]. Severe dental disease was found to be a risk factor for colic for working equids in Egypt, however giving anthelmintic in the preceding six months led to an increased incidence of colic [8]. It was noted in this study that the use of anthelmintics as a risk factor needed further evaluation, since this may be multi-factorial; anthelmintics may have been given in response to colic and the overuse of anthelmintics, and decreased efficacy due to the resistance of parasites needs consideration [8]. Evidence from other studies shows regular use of anthelmintics reduces the risk of colic [41,50], but recent administration may see an increased incidence [41]. In Honduras, the exact types of intestinal parasites are not known, and this requires further investigation to guide management practices and the targeted use, and not overuse, of anthelmintics [52]. The current study suggests that the use of preventative health care may be highly dependent on availability and accessibility for owners.

Changes in weather during different seasons has been described as a risk factor for colic. One veterinary practitioner reported an increased incidence of colic in the summer months, due to dehydration, feeding of concentrates, and equids scavenging and ingesting plastic bags. It was found in another study in working equids that there was an increased risk of colic in drier months [8]; studies in pleasure horses also show seasonality in both winter/colder [53] and hotter/summer months [54]. Regardless of location and climatic conditions, whether there are increased or decreased climatic temperatures, this seasonality is likely to occur due to a change in management practice that occurs when the season changes [41,53], and there is a need for information for owners targeted at specific high risk periods.

### 4.4. Knowledge and Understanding of Colic

In this study and two other studies in Ethiopia, it was found that owners consider colic an important condition in working equids [7,15]. In the study by Curran et al. [15], 59% of donkey owners reported rolling/colic/tympany in the previous year, and in the study by Stringer et al. [7], colic was ranked as the second most important problem by horse owners and ninth by donkey owners. In this study, owners had a varying understanding of colic, but out of the almost two thirds who knew what colic was, most could name more than one clinical sign. These clinical signs were sometimes different to those reported in previous studies. Previously reported indicators of abdominal pain include pawing, attempts to lie down, flank-watching, box-walking/circling, sweating, rolling, kicking, and subtle changes is demeanor/behavior, such as dullness, lowered head position, decreased response to the environment, and inappetence [27,28]. The signs of colic vary depending on the underlying etiology; for example, in large colon impaction, the signs of pain may be milder [55]. Similarly, signs of colic in donkeys are also considered more subtle [31,56,57].

In this study, the most common signs named by owners were in the ‘Restless or agitated’ group, followed by ‘rolling’ and ‘running’ also being mentioned. ‘Running’ is not a typically reported symptom, probably as most studies relate to horses that might be partially stabled, so an equivalent presentation might be ‘box walking’ [27]. The descriptions ‘could not stand’ were grouped into ‘Tired or lethargic’, but this is more dramatic than the other signs listed in this group. The clinical signs listed by Honduran owners are overtly obvious, and some of the more subtle signs were not listed at all. Only some owners mentioned less visible signs: eating less/nothing, less droppings, and being dull/quiet. Missing these subtle indications may mean some types/early stages of colic go unnoticed, such as with large colon impaction [55], and this is potentially more important in donkeys or mules which are often reported to not show obvious signs of pain [31,56]. In a study in Egypt, similar clinical signs were listed, with only one horse out of 460 showing a dull demeanor [8]. In an Ethiopian study on owner-reported equid diseases, it was considered that horse owners had a good knowledge of colic signs (rolling and restlessness) and donkey owners were able to describe more subtle signs (bloating, anuria, anorexia) [7]. All agrovets questioned in this study said that donkeys do not differ in their presentation of colic (5/5) and the three veterinary practitioners showed varying responses: with one providing a description of severe colic signs, one stating that colic is seen less often in donkeys, and one that donkeys are not particularly different from horses. The Donkey Sanctuary states: ‘detecting colic can be difficult and/or delayed in donkeys due to their stoic nature and the subtle clinical signs displayed’, with common signs listed as, ‘dullness, isolation from companion or group, reduced appetite and ineffective eating, recumbency’ [31]. Colic might be going unnoticed in donkeys until the more severe stages. Colic may therefore not be noticed in donkeys until the more severe stages. Veterinary practitioners and agrovets were able to recall obvious clinical signs of colic in horses (10/11), but a greater number of participants is required to gain a good understanding of their wider knowledge. This study suggests the need to review and compare how the presentation of colic may vary in working equids, and therefore how existing resources around colic need to be adapted for this population of horses.

### 4.5. Treatment of Colic

The most common treatment used by equid owners was a herbal treatment/laxative. This is different to the top colic treatments used by owners in Egypt: exercise and pharmaceutical preparations (with the top pharmaceutical used by owners being furosemide) [8]; however, these findings were similar to a study in Northern India, where home remedies were frequently used in colic cases [58]. Eighty percent were reported to recover from colic, which is similar to data from other studies [59]. None of the owners’ treatments (Table 3 are recognized colic treatments in the UK, except for hydration with water and electrolytes. Most treatments were implemented after some responded through trial and error and through asking a friend, an approach also reported in another study [58]. Some veterinary practitioners used laxatives in combination with other medical management for impactions [60,61], but more commonly hydration is a mainstay treatment for these cases via enteral fluids administered using nasogastric intubation [62]. It is important to consider, how many of these colic cases in the study area are impactions, and how many of these impactions are caused by dehydration due to the hot climatic conditions and hard workload; and therefore whether there is the possibility that laxatives may worsen the underlying cause. Re-hydration using water is a sensible option but only suggested by a quarter of owners. However, when equids are suffering from colic they are often unwilling to drink, which is one of the reasons why nasogastric intubation of fluids is often used by veterinary practitioners (first ensuring there is no excessive nasogastric reflux). It is also important to recognize the volume of fluids required to clinically resuscitate a clinically dehydrated animal, which is unlikely to be practical in many circumstances. Nasogastric intubation additionally has important therapeutic and diagnostic value for veterinary practitioners in cases of colic, to empty the stomach and assess contents, and to administer medication [27].

Ethnobotanical studies highlight the many uses of medicinal plants available in Latin America [63]. For example, Guacimo, the most commonly used treatment by owners, is the crushed seeds of the West Indian Elm (Guazuma ulmifolia). It is a common herbal remedy in Latin America, with many reported properties, including being anti-inflammatory, antimicrobial, and gastroprotective [64]. Curarina is curare, which is used in poison darts [65]. It is a nicotinic acetylcholine receptor (nAChR) antagonist causing muscle relaxation and death; however, as it has 0% bioavailability it is unlikely to cause harm if ingested. Ethnobotany has played an important role in drug discovery for many years [66], and whilst evidence for these medicinal plants’ use in equine colic is lacking, they may provide appropriate alleviation from colic signs.

Five owners used alcohol as a treatment, three forced exercise, seven gave antimicrobials, one fed cooking gas, and one fed meat juices from cooking, all of which may cause further harm to the animal. Two owners used more invasive techniques of rectal evacuation of plastic bags or percutaneous puncture of the intestines using a trocar, both of which are of significantly higher risk but potentially sensible options if given the correct training. The 6% that were treated by the veterinary practitioners at EQUHS were based locally to the site. Unfortunately, this is not an option for the more remote areas, so educational resources are clearly needed.

Decision-making practices are complex [58], and in many cases, remedies readily available in communities should not be discouraged without reason, so long as equid welfare is not compromised. However, with the wide variations of treatments used, even between small communities in Honduras, it would be challenging to research which home remedies have positive (or negative) impacts in a wider population. Overall, the owners visiting the clinics all have an interest in their equids’ health, and there are still many more that are not reached by EQUHS or other veterinary teams. These owners may be carrying out different practices in addition to those documented in this study.

### 4.6. Observations on Differences between Honduras and the UK Veterinary Drugs and Veterinary Practice

In this study, many of the owners would perform their own homemade remedies first, and rarely call a veterinary practitioner, and more commonly the agrovet. In both the UK and Honduras, veterinary care can be cost prohibitive, and in a LMIC where the owners are often living in poverty this is more significant. There are no equine hospitals in Honduras, and colic surgery cannot be performed in the field due to sterility; furthermore, this would be costly and high risk for a low value equid. Typical euthanasia drugs available in the UK such as Somulose are not available in Honduras. Furthermore, these animals are essential for people’s livelihoods; therefore, electing to euthanize was infrequent.

There is no evidence to suggest diuretics are a suitable drug for the treatment of colic (given by half of the agrovets questioned), and the only indications in pleasure/performance horses are for treating exercise-induced pulmonary hemorrhage, acute renal failure, and congestive heart failure [67]. Dehydration is a common finding in the working equid population [44,45], which can lead to anuria, commonly reported as ‘urine retention’ [8], and this may be the reason why a diuretic is prescribed, so that the equid urinates. Another possible reason why this drug is given is that some equids with abdominal pain adopt an antalgic position, such as stretching and posturing to urinate, without urination [27,29]. The reasons for choice of furosemide were not explored in this study, but it is likely due to misunderstanding of the condition and the reason why the equid is failing to urinate. This treatment was also commonly recognized for use by veterinary practitioners and owners in another study in Egypt [8].

Although not mentioned in the questionnaire, some owners reported agrovets to recommend antimicrobials, most commonly oxytetracycline in the treatment of colic. Colic rarely has a bacterial etiology, so the use of these classes of drugs may lead to increasing antibiotic resistance, especially when combined with inappropriate dosing. The issue of antimicrobial resistance was highlighted in another study exploring drug retail outlets in northern India [68], with lack of training considered a key area for improvement.

When asked, ‘When would you see the vet?’, 25% of owners reported they would go to the agrovet, 22% said they would treat first and only go if their treatments did not work, and 17% said they would see the veterinary practitioner if their animal was unwell. It was only part-way through the study that it was recognized that owners used the term vet to refer to both agrovets and fully qualified veterinary practitioners. Subsequently, this question was made more specific, and these results are likely to be an inaccurate representation of the question as intended. The main barriers to veterinary treatment reported were accessibility and the cost. The decision to self-treat before seeking veterinary assistance may also be driven by community and personal factors, alongside economic and infrastructural considerations [58].

### 4.7. Study Strengths, Limitations, and Future Research

Efforts were taken to reduce bias within the study design; however, it is likely bias remains. Convenience sampling was used and whilst the study population covered 11 communities in which EQHUS worked, and with varying topography, the nature of the sampling and small sample size means deeper exploration of the data using hypothesis testing is inappropriate. There will be selection bias since only the Choluteca region of Honduras was visited. Furthermore, questionnaire participants were accessed via equid owners/keepers engaged in community visits, and so may have been more motivated than the general equid owning population and have periodic access to veterinary treatment and education. There will be seasonal bias as the researcher visited in June, when water and food is abundant and there are reportedly fewer colic cases. No colic cases were observed by the researcher within the data collection period. There will be some information bias where cultural/translation differences may affect data analysis. In this study, the responses from the veterinary practitioner and agrovet population were mainly used to develop an understanding of the environment and context.

Further baseline data is required from Central America on owners, veterinary practitioners, and agrovets for triangulation of the data in this study and the development of educational resources. Recommendations for future work include repeating the study in other populations (both in Honduras and other countries), qualitative studies with groups of veterinary practitioners and agrovets within a wider population, and a specific focus to identify barriers to change amongst equid owners/caregivers. Following this, interventions can be made and progress closely followed using monitoring and evaluation [69]. Improving access to knowledge on husbandry practices does not necessarily lead to changes of behavior that improve equine welfare. The theories surrounding behavioral change need to be considered, and a strong understanding of beliefs and values as a baseline are critical before any attempts to implement change [70,71,72].

## 5. Conclusions

This study provides an overview of the understanding of colic within the study population, and highlights keys areas for future work and understanding. There are clearly distinct approaches and risk factors for equine colic, compared to countries and regions where research has been previously undertaken. The knowledge of colic is variable, with owners, veterinary practitioners, and agrovets all citing different approaches. Fundamentally, the majority of individuals desired to do something for the equids, whether it be using home remedies or seeking an agrovet or qualified veterinary practitioner.

This exploratory study has highlighted a number of areas for targeted educational interventions to impact the risk of poor welfare in working equids. The key areas identified are: the understanding of what colic means, recognition and actions to reduce the risk of colic, and knowledge of appropriate treatments and sources of information. Proven, cost-effective interventions can transform equine welfare by providing primary veterinary care and community training, which can reach tens of thousands of equids within the programs. 

## Figures and Tables

**Table 1:** Descriptions of communities where the owner questionnaires were carried out. Field notes related to each community are presented in Supplementary Materials (Table S1). * Asterisk denotes data for approximate total number of equids and owners registered to EQHUS in these areas.

Community	Number of Owners Interviewed	Types of Equid	Number of Equids	Approx. Total Number of Equids *	Approx. Total Number of Owners *	Notes
Thelmo Ruiz	11	Horses	23	55	50	Urban. Near EQHUS office/clinic in the valley
Las Piletas	5	Horses	11	N/A	N/A	Urban. Near EQHUS office/clinic in the valley
Santa Maria	4	Horses	6	N/A	N/A	Urban. Near EQHUS office/clinic in the valley
Tapatoca	5	Horses, mules	22	50	43	Rural and mountainous
El Carrizal’ Trapiche	12	Horses, donkeys, mules	35	110	92	Rural and mountainous
Apinto El Corpus	11	Horses, donkeys, mules	26	120	100	Rural and mountainous
Brisas del Rio	4	Horses	8	N/A	N/A	Urban. Near EQHUS office/clinic in the valley
El Eden	11	Horses	23	N/A	N/A	Urban. Near EQHUS office/clinic in the valley
La Puente	8	Horses	18	65	50	Urban. Near EQHUS office/clinic in the valley
Trapiche	12	Horses, donkeys, mules	25	110	92	Rural and mountainous
San Lorenzo	10	Horses	14	160	155	Urban and coastal

**Table 2:** Clinical signs of colic listed by 65 owners in Choluteca, Honduras during verbal questionnaires and grouped according to the ‘REACT’ acronym [27] (Supplementary Items, Figure S1). Number indicates the total number of owners that gave this response, which were totaled, with a percentage given to each symptom. A total of 181 clinical signs were given, i.e., some owners mentioned more than one clinical sign.

Signs	Signs	Number	Percentage
Restless or agitated	Rolling	58	32%
	Lying down and getting up	17	9%
	Running	16	9%
	Fall down/throw to the floor	11	6%
	Unexplained sweating	1	<1%
Eating less or droppings reduced	Eating and drinking less/nothing	10	6%
	Passing less/no droppings	4	2%
Abdominal pain	Flank watching	11	6%
	Kicking at abdomen	7	4%
	Stretching	4	2%
	Biting abdomen	1	<1%
Clinical changes	Abrasions/wounds	2	1%
Tired or lethargic	Could not stand	19	10%
	Dull/quiet	4	2%
	Increased lying down	1	<1%
Other	‘Pain’	7	4%
	Bloat/tympanism	3	2%
	Blindness	2	1%
	Running into things	1	<1%
	Gets mad	1	<1%
	Biting wood	1	<1%
Total		181	100%

**Table 3:** Treatments of equids with colic listed by 67 owners in Choluteca, Honduras during verbal questionnaires. Number indicates the total number of owners that gave this response, which were totaled, and a percentage given to each symptom. A total of 162 treatments were mentioned, i.e., some owners mentioned more than one treatment they use for colic. Guacimo, hombre grande, cow seed, thalmaltisco, cucaracha trunk, gutya, and curarina all refer to local herbal remedies.

Treatment Type	Specific Treatment	Number	Percentage
Herbal treatment/laxative	Guasimo	34	21%
	Garlic	17	10%
	Curarina	10	6%
	Thalmaltisco	3	2%
	Cucaracho bark	2	1%
	Hombre Grande	2	1%
	Tobacco	2	1%
	Cow seed	1	<1%
	Sugarcane	1	<1%
	Gutya	1	<1%
	Coffee	1	<1%
Water	Mixed with salt/with salt and other ingredients	17	10%
	Mixed with other ingredients (not salt)	7	4%
	On its own	2	1%
‘Other’ treatment	Vinegar	10	6%
	Oil	8	5%
	Cooking gas	1	<1%
	Epsom salts	1	<1%
	Meat juices from cooking	1	<1%
	Salt (water not mentioned)	1	<1%
	Food	1	<1%
	Bicarbonate	1	<1%
Medical treatment	Unknown injection/pill	9	6%
	Oxytetracycline/antibiotic	7	4%
	Spasmolytic	2	1%
	Nasogastric intubation and fluids (given by veterinary practitioner)	1	<1%
	Piriton	1	<1%
	Anthelmintics	1	<1%
Physical treatment	Abdominal massage/with stick	5	3%
	Running them	3	2%
	Wash with cold water	2	1%
Alcoholic drink	Beer	3	2%
	Schnapps/liquor	2	1%
Invasive treatment	Rectal exam and plastic bag removal	1	<1%
	Trocar	1	<1%
Total		162	100%

## Data Availability

Anonymized, raw data is available. DOI: 10.17639/nott.7120.

## Supplementary Materials

The following are available online at https://www.mdpi.com/article/10.3390/ani11072087/s1, File S1: Equid Owner Colic Questionnaire, Figure S1: The British Horse Society REACT acronym, Table S1: Community profiles.
