# Supplementary material for: Owners’ Knowledge and Approaches to Colic in Working Equids in Honduras
Source: Animals (Basel). 2021 Jul 13;11(7):2087. doi: 10.3390/ani11072087 (PMC8300094; doi:10.3390/ani11072087)
Supplement: Supplementary file 1 [file animals-11-02087-s001.zip › animals-1271642-supplementary.pdf]

Supplementary Items

Supplementary Items 1 – File S1. Equid Owner Colic Questionnaire (translated to Spanish)

* Asterisk denotes questions where multiple answers were encouraged and documented in the results.

General

1. Male/Female
2. Number of equids owned
3. Type of equids – donkey/mule/horse
4. Type of work your equid does (main work) *
   Examples: Firewood collection, collecting recycling materials, transportation of people, other (to be developed in Honduras)
5. Number of hours per day worked
6. Number of days working per week

Colic understanding

1. Do you know what colic is? – yes/no
2. What signs of colic do you know? *
   Examples: reduced appetite, does not eat, does not drink, no droppings, refuses to move, lies on ground, rolls on ground, pawing at ground, kicks/bites belly, looking at belly (flank watching), working less, bloated, sweating, stretching
3. What causes of colic do you know? *
   Examples: no water, bad food, too much food, eating rubbish, too much food, too much work, heat, drinking after hard work, cold water, change in diet, microbe
4. Have you experienced colic before? – yes/no
5. What did you do? *
   Examples: nothing, treat yourself (how?), rest animal, ask a friend, take to vet or paraprofessional, other
6. Did the equid recover from colic? – yes/no
7. When would you ask for the help of a vet?
   Examples: never, eats less, does not eat, works less, refuses to move, lies on ground, emergency

Management

1. How many times a day does your equid have access to water?
2. Where is the water from?
3. How many times a day is the equid fed/have access to feed?
4. What do you feed your equid?
   Examples: grass, grass + concentrates, concentrates
5. Do you ever change what you feed your equid? – yes/no
6. Do you deworm your equid? – yes/no
7. Routine dentistry/teeth rasping? – yes/no
8. Where does the equid live?
   Examples: loose, stabled, tied up
9. Does your equid have access to rubbish unattended? – yes/no

Supplementary Items 2 – Figure S1

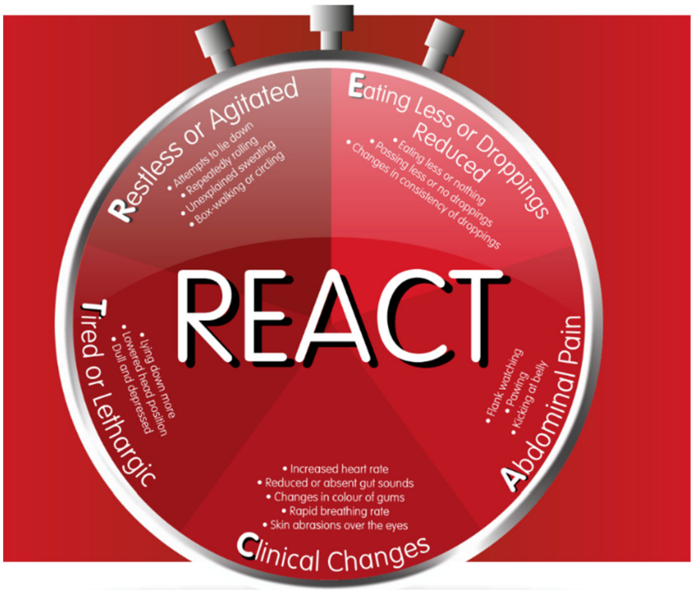

Supplementary Items 2 – Figure S1. The British Horse Society REACT acronym [26], indicating common equine colic sign(s) seen in the UK. This acronym was used to group the sign(s) listed by 65 working equid owners in Choluteca, Honduras from a cross-sectional study on experiences, approaches and knowledge of colic.

**R**estless or agitated	**E**ating less or droppings reduced	**A**bdominal pain	**C**linical changes	**T**ired or lethargic
• Attempts to lie down<br>• Repeated rolling<br>• Unexplained sweating<br>• Box-walking or circling	• Eating less or nothing<br>• Passing less or no droppings<br>• Changes in consistency of droppings	• Flank watching<br>• Pawing<br>• Kicking at belly	• Increased heart rate<br>• Reduced or absent gut sounds<br>• Changes in colour of gums<br>• Rapid breathing rate<br>• Skin abrasions over eyes	• Lying down more<br>• Lowered head position<br>• Dull

Supplementary Items 3 – Table S1

Community	Number of owners	Types of equid	Notes
Thelmo Ruiz	11	Horses	This community was located near the EQUHS office/clinic in the valley. The most common work was firewood collection (7). Skin conditions were the main problem observed with ectoparasites. Other common problems seen by the vet were wounds, lameness, respiratory conditions and eyes. People were happy to be interviewed, and most had an understanding of colic (10) and mainly gave their own treatments (8), used the agrovet (2) or did nothing (2). Treatments were: salt and water (4), garlic (8), guacimo (2), vinegar (2), abdominal massage (2), product from the agrovet (2), cooking gas (1), oxytetracyline (1), spasmolytic (1). Owners reported that some got better, others not. Dentistry/teeth rasping had been carried out in this community (5). Reported increase in colic in the summer. One castration was carried out.
Las Piletas	5	Horses	This community was not far from Thelmo Ruiz. Similar problems were observed here. Horses were generally in good condition. They were all used for (or will be used for) firewood collection. 3/5 knew what colic was and were able to name somesigns, but only 2 knew causes of colic. Two used their own treatments (salt and water +/- guacimo), 1 used the agrovet (antibiotics). One castration was carried out.
Santa Maria	4	Horses	Again this community was not far from the EQUHS office/clinic, located in the valley. Horses were generally in good condition. Most owners had a good knowledge of colic and what they would do. Two of the interviews were slightly encrypted but most 2 equids were used firewood collection and 1 for carrying corn. They had all had prior experience of colic. Treatments were: using mineral oil and a plastic bag as a glove to clear rubbish from the rectum and feeding leftover juices from cooking meat as a laxative.
Tapatoca	5	Horses mules	A rural community up in the mountains. Here the animals were mainly used for dairy processes and with the cattle and mules are used alongside horses. The view was that donkeys are stronger and mules even stronger so can be worked harder. Many of the horses were not castrated as they were perceived to lose their strength. A few horses were being used for parades/shows only. Some had some very overgrown feet and harness wounds. There was less rubbish in the area compared to the more urban areas. All owners were able to name signs of colic, but only one was able to name causes. Most used their own treatments, with one using an agrovet. The treatments were guacimo (3), salty water (3), vinegar (3), agrovet (oxytetracyline, diparona) (1), cow seed (1), running (1), beers (1). We also

			interviewed the agrovet here, the business was part of a government scheme to encourage small business development in rural areas, it was an agrovet and general store combined. He received a lot of advice from agrovets in Choluteca and also the government organisation. The agrovet was aware of some of the signs of colic but not the risks.
El Carrizal' Trapiche	12	Horses Donkeys	A rural community in the mountains – difficult to access and horses are essential for transportation due to poor infrastructure. There were over 60 horses, mules and donkeys which turned up belonging to about 12 owners (who had workers working for them). The equids were used for a variety of jobs – transportation of people, collecting firewood, and transporting maize and crops. The equids were in good condition, especially the donkeys and mules and there was not much rubbish. There were a number of mares with foals at foot. There were many stallions but the owners did not want to castrate. Six knew what colic was, 7 could name signs and only 4 could name a cause. Five would treat themselves and one went to SENASA (government vets) for treatment. The treatments were: guacimo (7), curarina (5), salt water (2), massage with stick (2), water (1), vinegar (2), liquor (1), beer (1).
Apinto El Corpus	11	Horses<br>Donkeys<br>Mules	A welcoming remote and mountainous community with a mix of older and younger generation equid owners/keepers attending. Horses, mules and donkeys were mainly used for agriculture (7), transportation/riding (8) and firewood collection (2). They were in fairly good condition, but the mares with foals underweight. Seven owners knew what colic was, 10 were able to name signs and 7 able to name causes. Most were vocal on parasites as a cause of colic and thanked the EQUHS group for a possible reduction in colic since the ivermectin treatments. Most had experienced colic (10), 9 treated themselves and 1 went to the vet. Treatments in this community had a large variation of herbal remedies: guacimo (4), hombre grande (2), garlic (6), thalmaltisco (3), cucaracha trunk (2), sugarcane (1), gutya (1), curarina (2). Alcoholic remedies were also used: beer (1), liquor/schnapps 1 litre (1). Other remedies: stick (1), oil (3), vinegar (1), running them (1), salt and water (3), piriton (1). Some had homemade parasite treatments. One explained the older generation of men gave the recipe for treatments.
Brisas del Rio	4	Horses	This was a small community visit near to the EQUHS office/clinic in the valley. Here more women were interviewed than men. The only horses were present and used for firewood collection (2) and cattle work (2). The owners all knew what colic was, could name signs and 3 could name causes. All 4 had experienced colic before – with 2 more than once, 3 treated themselves, one asked a neighbour and one tried to make contact with EQUHS but did not answer. The treatments were guacimo (3), salt (2), oil (2), water (1), vinegar (1).

El Eden	11	Horses	This community was near to the EQUHS office/clinic, based in the valley. Some of the owners used EQUHS veterinary staff with success stories, others used the agrovet. The community owned horses only, which were used for firewood collection (7) and rubbish collection (3). Six owners knew what colic was, 8 could name signs, 7 could name possible causes. Eight owners had experienced colic before, 4 treated themselves, 3 used EQUHS and 2 used the agrovet. Treatments were: guacimo (5), garlic (3), salt and water (1), Epsom salts (1), nasogastric tube and fluids (1) and unsure of treatment (3). Five had dentistry/teeth rasping by the Equitarians (an American charitable veterinary group).
La Puente	8	Horses	This community was based in the valley. Only horses were used for carrying firewood (5), riding (2) and carrying water (2). Five owners knew what colic was, 5 could name signs, 3 could name causes. Four had experienced colic before, once three times. 3 treated themselves, 1 used the agrovet. Treatments were: vegetable oil (2), oxytetracyline (2), guacimo (1), garlic (1), trocar (1). Dentistry/teeth rasping was carried out for 2 horses.
Trapiche	12	Horse<br>Donkeys<br>Mules	This community was in the mountains, with equids used mainly for riding (10), carrying water (2), carrying milk (1), carrying corn (1), cattle (2), breeding (1). Eight owners knew what colic was, 9 could name signs, 7 could name causes. Nine owners had experienced colic before (1 frequently), 8 treated themselves, 1 did nothing. Treatments were guacimo (6), tabacco (2), running them (2), garlic (2), water (2), corarima (2), oxytetracyline (1).
San Lorenzo	10	Horses	This community was based by the coast. The horses were mainly used for carrying/selling water (7), followed by firewood (4). Only 3 owners knew what colic was, 4 could name signs and 3 could name causes. Five owners had experienced colic before, 5 treated themselves. The treatments were oxytetracyline (2), bath of salt and water (2), giving water and resting (1), giving food (1), deworm (1), pill unknown (1), salt and water drink (1), coffee (1). There was history of dentistry/teeth rasping for one horse. In this community an agrovet was also interviewed.

Supplementary Items 3– Table S1. Community profiles from a cross-sectional study of Honduran working equid owners' experiences, approaches and knowledge of colic. The numbers in brackets refer to the number of owners who gave this response. Guacimo, hombre grande, cow seed, thalmaltisco, cucaracha trunk, gutya and curarina all refer to local herbal remedies.
